# Comparative efficacy of levofloxacin, azithromycin, and doxycycline prophylaxis and treatment in an experimental *Ureaplasma* murine lung infection model

**DOI:** 10.1128/aac.01724-24

**Published:** 2025-04-14

**Authors:** Derek Fleming, Maha Y. Al-Jabri, Robin Patel

**Affiliations:** 1 Division of Clinical Microbiology, Department of Laboratory Medicine and Pathology, Mayo Clinic 195112 , Rochester, Minnesota, USA; 2 Division of Public Health, Infectious Diseases, and Occupational Medicine, Department of Medicine, Mayo Clinic 4352, Rochester, Minnesota, USA; University of California San Francisco, San Francisco, California, USA

**Keywords:** *Ureaplasma*, antibiotics, lung infection, antibiotic resistance

## Abstract

Although lung transplant recipients who develop *Ureaplasma-*associated hyperammonemia syndrome are treated with *Ureaplasma*-targeted antibiotics, optimal therapy is incompletely defined, and typically not based on real-time antimicrobial susceptibility testing results. This study compared levofloxacin, azithromycin, and doxycycline prevention and treatment of *Ureaplasma urealyticum* (UU) and *Ureaplasma parvum* (UP) infections in an immunosuppressed murine lung infection model. For prophylaxis, mice received antibiotics before infection with UP-FS (susceptible to all study antibiotics), UP-AzmR (azithromycin-resistant), UP-LevR (levofloxacin-resistant), or UU-DoxR (doxycycline-resistant), with lung bacterial loads (color changing units [CCUs]) measured 18 hours following infection. For UP-FS, doxycycline was most active (Δ4.84 log_10_ CCU/g; *P* = 0.0006), followed by levofloxacin (Δ2.45 log_10_ CCU/g; *P* = 0.018), with azithromycin yielding a nonsignificant CCU reduction. Doxycycline (Δ1.9 log_10_ CCU/g; *P* = 0.0025) and levofloxacin (Δ1.3 log_10_ CCU/g; *P* = 0.004) showed activity against UP-AzmR. Only doxycycline showed activity against UP-LevR (Δ2.28 log_10_ CCU/g; *P* = 0.0002), and only azithromycin showed activity against UU-DoxR (Δ0.67 log_10_ CCU/g; *P* = 0.0175). For treatment, antibiotics were administered 24 hours following infection; doxycycline significantly reduced bacterial loads of all study isolates, except UU-DoxR. In addition, azithromycin was active against UP-LevR (Δ1.21 log_10_ CCU/g reduction; *P* = 0.0003). In summary, except for doxycycline-resistant UU, doxycycline was active in preventing and treating *Ureaplasma* lung infection in immunosuppressed mice.

## INTRODUCTION

Nearly 3,000 lung transplants are performed every year in the United States (1), with numbers anticipated to grow as availability and survivability continue to improve. An important contributor to survivability has been the minimization of mortality associated with post-transplant infections. Hyperammonemia syndrome has historically occurred in ~4% of lung transplant recipients (2, 3). Ammonia (NH_3_) crosses the blood–brain barrier and is converted to glutamine and glutamate, leading to mitochondrial impairment, depletion of 2-oxoglutarate, disruption of the tricarboxylic acid cycle, death of glial cells, and ultimately, cerebral edema (4, 5). In lung transplant recipients, clinical manifestations progress from altered mental status to seizures, coma, and often death (6–10). Hyperammonemia syndrome in lung transplant recipients is associated with infection with *Ureaplasma* species, which are normally considered commensal microbiota of the urogenital tract. These bacteria produce a potent urease that splits urea into NH_3_ and CO_2_ as a means of ATP synthesis (11–13). Two human-associated *Ureaplasma* species, namely, *Ureaplasma urealyticum* (UU) and *Ureaplasma parvum* (UP), have been linked to hyperammonemia syndrome, with evidence for donor transmission (14–16). This discovery has led to improvements in the outcomes of patients with hyperammonemia syndrome, including prophylaxis and treatment with *Ureaplasma*-directed antibiotics (17, 18).

While *Ureaplasma*-directed antibiotics have reduced morbidity and mortality, ideal treatment has not been defined. *Ureaplasma* species lack a cell wall, rendering cell wall-targeting antibiotics (β-lactams and glycopeptides) ineffective. They do not synthesize folic acid, making sulfonamides and diaminopyrimidines inactive. Nucleic acid replication inhibitors (e.g., fluoroquinolones) and protein synthesis inhibitors (e.g., tetracyclines and macrolides) have activity against *Ureaplasma* species (13). However, acquired resistance to macrolides, tetracyclines, and fluoroquinolones has been reported (12, 17), including the emergence of resistance during therapy (18). Today, results of antimicrobial susceptibility testing are rarely available in a clinically useful time frame.

Here, an immunosuppressed mouse model of *Ureaplasma* respiratory tract infection was used to investigate the activity of doxycycline, azithromycin, and levofloxacin against *U. parvum* or *U. urealyticum* resistant to one of the study antibiotics, as well as a control *U. parvum* isolate susceptible to all three study antibiotics.

## MATERIALS AND METHODS

### Study isolates

Study isolates are shown in Table 1. Doxycycline, azithromycin, ciprofloxacin, tetracycline, erythromycin, and levofloxacin minimum inhibitory concentrations (MICs) were determined in duplicate using broth microdilution, following the Clinical and Laboratory Standards Institute guidelines (19). A range of antimicrobial concentrations from 0.125 to 16 µg/mL was used in 10B broth (Remel, Lenexa, KS). *U. urealyticum* American Type Culture Collection (ATCC) 33175 served as a quality control isolate; it also served as the doxycycline-resistant infection isolate (UU-DoxR) in the experiments described below.

**TABLE 1:** Bacterial isolates studied

Isolate	Abbreviation	Source	Resistance	Resistance mechanism
*U. parvum* IDRL-10774	UP-FS	BAL fluid	None	N/A^*a*^
*U. parvum* IDRL-10774R	UP-AzmR	BAL fluid	Azithromycin	Unknown
*U. parvum* IDRL-11160	UP-LevR	Urine	Levofloxacin	Δ*parC* (20)
*U. urealyticum* ATCC 33175	UU-DoxR	Urine	Doxycycline	*tetM* (21)

^*a*^ N/A, not applicable.

### Selection of macrolide resistance in *U. parvum* IDRL-10774

**Fig 1:**
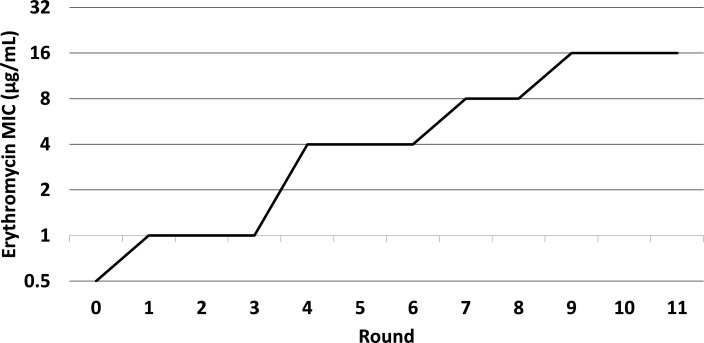
Experimental evolution of macrolide resistance in *Ureaplasma parvum* IDRL-10774. *U. parvum* IDRL-10774 was serially passed through 75% MIC concentrations of erythromycin in a dialyzed flow system to evolve resistance. Resulting MICs were measured after each round.

*U. parvum* IDRL-10774 (UP-FS), a clinical bronchoalveolar lavage (BAL) isolate susceptible to doxycycline, azithromycin, and levofloxacin, was serially passed in sub-MIC erythromycin concentrations in a dialyzed flow system (22). The susceptibility of *Ureaplasma* species to erythromycin and azithromycin is considered equivalent (19). UP-FS was inoculated into a 10 mL dialysis tube (Spectra/Por Float-A-Lyzer G2 1,000 kD Dialysis Device; G235073) and submerged in the flow chamber with 200 mL of 50 mM MES-buffered U9 media (23) containing erythromycin at 75% of its MIC (19). The MIC of the starting isolate was 0.5 µg/mL; the MIC was recalculated after each round. The flow rate of antibiotic-containing media was 2 mL/hour (1% per hour). After 48 hours of flow, 1 mL was frozen for antibiotic susceptibility testing (AST), and 1 mL was used for color-changing unit (CCU) quantification in fresh 10B broth (Remel R20304). CCUs were quantified in triplicate for 72 hours, after which frozen aliquots were thawed, spun down, resuspended in 10B broth, and diluted to 10^4^ CCU/mL in fresh 10B for AST. Flow was conducted sequentially using each new MIC; if 75% MIC resulted in no growth, 68% and subsequently 50% MIC (if needed) were tested. Serial flow experiments were repeated until a MIC ≥16 µg/mL was achieved (Fig. 1). The resulting macrolide-resistant strain is denoted as *U. parvum* IDRL-10774R (UP-AzmR).

### Mouse model of *Ureaplasma* lung infection

C3H male and female mice (18–22 g, Charles River Laboratories, Wilmington, MA) were pharmacologically immunosuppressed for 7 days using methylprednisolone, tacrolimus, and mycophenolate mofetil, mimicking a typical immunosuppressive regimen in lung transplant recipients. Animals were administered 40 g/L urea *ad libitum* in their drinking water beginning 7 days before infection and/or treatment. For inoculum preparation, frozen *Ureaplasma* aliquots were thawed and cultured in U9 broth (23) at 37°C in a 5% CO_2_ incubator until the media color shifted from yellow to orange. The bacterial suspension was then centrifuged at 12,500 rcf for 30 minutes to pellet the bacteria, which were subsequently resuspended via pipette mixing and vortexing in saline with 0.1% agar (to increase viscosity and reduce expulsion from the respiratory tract) (15, 16) to achieve concentrations of 10^7–8^ CFU/mL. For intratracheal (IT) challenge, mice were anesthetized with ketamine/xylazine (90/10 mg/kg); 50 µL of 10^7–8^ CFU/mL bacterial suspension was placed into their trachea using a 22G curved gavage needle, after which the animals were placed in a vertical position for 10 minutes using a murine vertical stabilization apparatus (22, 24). A combined IT/intraperitoneal (IP) challenge was utilized since it is more likely to produce a hyperammonemic state than either IT or IP administration alone, although the resulting extrapulmonary bacterial load remains low (15, 16). For the IP challenge, 100 µL of 10^7–8^ CFU/mL bacterial suspension was injected into the peritoneum. For prophylaxis, mice were administered systemic antibiotics 1 hour prior to infection and euthanized 18 hours later (without subsequent antibiotic doses). The lungs were harvested for lung CCU quantification (log_10_ CCU/g lung tissue), which was tested in triplicate. The limit of detection (LOD) was equal to the log_10_ of 1 CCU divided by the lung weight (e.g., for lungs weighing 0.120 g, LOD = log_10_1.0 CCU/0.120 g = 0.92 log_10_ CCU/g). For postinfection treatment, mice were infected with a study *Ureaplasma* isolate and, 24 hours later, treated with systemic antibiotics and reinfected due to the short-lived nature of infection in this model. Eighteen hours following treatment, the animals were euthanized for lung CCU quantification in U9 broth, in triplicate. Ammonia concentrations (µmol/L) were measured in whole blood immediately after cardiac puncture using an Arkray PocketChem BA PA-4140 meter (ARKRAY America, Inc., Minneapolis, MN).

### Antibiotic dosing

The azithromycin dose was previously established as 100 mg/kg orally (PO) every 12 hours (25). For doxycycline and levofloxacin, pharmacokinetic profiles in mice were performed to inform treatment dosing for respiratory tract infections (area under the curve to minimum inhibitory concentration [AUC/MIC] ≥24 for doxycycline [26, 27] and ≥30 for levofloxacin [28, 29]). Doxycycline hyclate was administered IP every 12 hours for three doses, and blood was collected at 0.5, 1, 2, 4, and 6 hours after the final dose. Levofloxacin was administered subcutaneously (SQ) every 12 hours for three doses, and blood was collected at 0.5, 1, 2, and 4 hours after the final dose. Three animals were studied for each time point. Blood was collected via cardiac puncture at euthanasia, and serum was separated by centrifugation. Bioassays were performed using drug diffusion from 5 mm wells on Mueller–Hinton agar plates seeded with *Bacillus cereus* (ATCC 10987) for doxycycline and *Bacillus subtilis* (ATCC 6633) for levofloxacin, with zones of inhibition compared to a standard curve to determine serum concentrations. The determined dosing for doxycycline was 100 mg/kg IP every 12 hours, achieving an AUC/MIC of 41. For levofloxacin, a dose of 100 mg/kg SQ every 12 hours was selected, achieving an AUC/MIC of 30.

## RESULTS

### Prophylaxis

**Fig 2:**
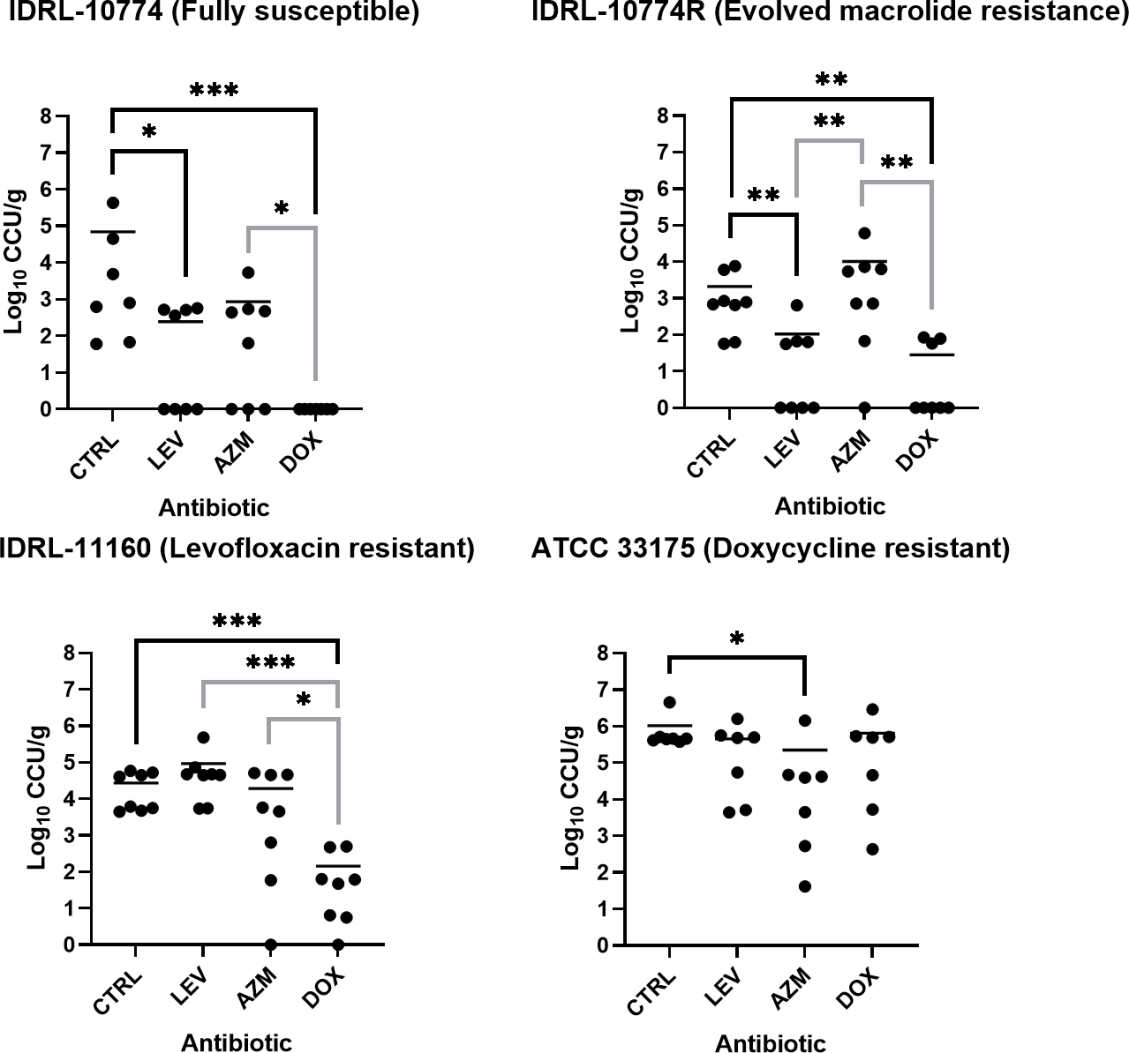
Antibiotic prophylaxis of murine infection by antibiotic-resistant *Ureaplasma* isolates. Mice received prophylaxis with systemic azithromycin (AZM), levofloxacin (LEV), or doxycycline (DOX) prior to infection with *Ureaplasma parvum* IDRL-10774 (susceptible to levofloxacin, azithromycin, and doxycycline), *U. parvum* IDRL-10774R (evolved macrolide resistance, susceptible to levofloxacin and doxycycline), *U. parvum* IDLR-11160 (levofloxacin-resistant, susceptible to doxycycline and azithromycin), or *Ureaplasma urealyticum* ATCC 33175 (doxycycline-resistant, susceptible to levofloxacin and azithromycin). Lung bacterial loads (log_10_ CCU/g) were quantified 18 hours after infection. Each prophylaxis group was compared to no-prophylaxis controls (black significance lines) and to one another (gray significance lines). Horizontal lines indicate the mean. Significance was determined via Mann–Whitney tests. *N* = 7–8. *P ≥ 0.05, **P ≥ 0.01, ***P ≥ 0.001.

To test the ability of antibiotic prophylaxis to protect against *Ureaplasma* lung infection in mice, animals were administered azithromycin, levofloxacin, or doxycycline 1 hour prior to infection with *Ureaplasma* isolates resistant to one or none of the antibiotics. Eighteen hours following infection, animals were euthanized, and lung bacterial loads were quantified. Results are shown in Fig. 2. Levofloxacin and doxycycline reduced the lung bacterial burden of UP-FS, a clinical isolate susceptible to all three tested antibiotics, with doxycycline exhibiting greater activity (mean reduction, 4.84 log_10_ CCU/g *versus* control; *P* = 0.0006) than levofloxacin (mean reduction, 2.45 log_10_ CCU/g *versus* control; *P* = 0.018). Azithromycin prophylaxis of UP-FS resulted in an insignificant reduction (1.19 log_10_ CCU/g; *P* = 0.07); however, 3/8 (37.5%) of azithromycin-prophylaxed mice had lung bacterial loads below LOD (<1 log_10_ CCU/g) *versus* 0/7 (0%) animals not receiving prophylaxis, 4/8 (50%) of levofloxacin-prophylaxed mice, and 7/7 (100%) of doxycycline-prophylaxed mice (Table 2). *U. parvum* IDRL-10774, the isolate with evolved macrolide resistance (UP-AzmR; IDRL-10774R), was also reduced by levofloxacin (mean reduction, 1.3 log_10_ CCU/g *versus* control; *P* = 0.004) and doxycycline (mean reduction, 1.9 log_10_ CCU/*g versus* control; *P* = 0.0025), while azithromycin was not active. None of the eight non-prophylaxed mice infected with UP-AzmR had lung bacterial loads below the LOD, compared to half (4/8) in the levofloxacin group, 12.5% (1/8) in the azithromycin group, and 62.5% (5/8) in the doxycycline group. For the levofloxacin-resistant *U. parvum* isolate (UP-LevR; IDRL-11160), only doxycycline was active as prophylaxis (mean reduction, 2.28 log_10_ CCU/*g versus* control; *P* = 0.0002); doxycycline and azithromycin prophylaxis for UP-LevR each had single animals with lung bacterial loads below LOD. For the doxycycline-resistant isolate (UU-DoxR), azithromycin was the only active prophylactic antibiotic (mean reduction, 0.67 log_10_ CCU/*g versus* control; *P* = 0.0175), and no mice had lung bacterial loads below LOD for any group. No hyperammonemia was detected in any animal.

**TABLE 2:** Percentage of infection in mice prophylaxed with levofloxacin, azithromycin, or doxycycline and infected with each of the four study *Ureaplasma* isolates

Infection isolate	Resistance	Percent of infection per prophylactic antibiotic							
		No antibiotic		Levofloxacin		Azithromycin		Doxycycline	
		%	*P*	%	*P*	%	*P*	%	*P*
UP-FS	None	100% (7/7)	NA	50% (4/8)	0.077	62.5% (5/8)	0.20	0% (0/7)	0.0006
UP-AzmR	Azithromycin	100% (8/8)	NA	50% (4/8)	0.077	87.5% (7/8)	>0.99	37.5% (3/8)	0.026
UP-LevR	Levofloxacin	100% (8/8)	>0.99	100% (8/8)	>0.99	87.5% (7/8)	>0.99	87.5% (7/8)	>0.99
UU-DoxR	Doxycycline	100% (7/7)	>0.99	100% (7/7)	>0.99	100% (7/7)	>0.99	100% (7/7)	>0.99

### Treatment

**Fig 3:**
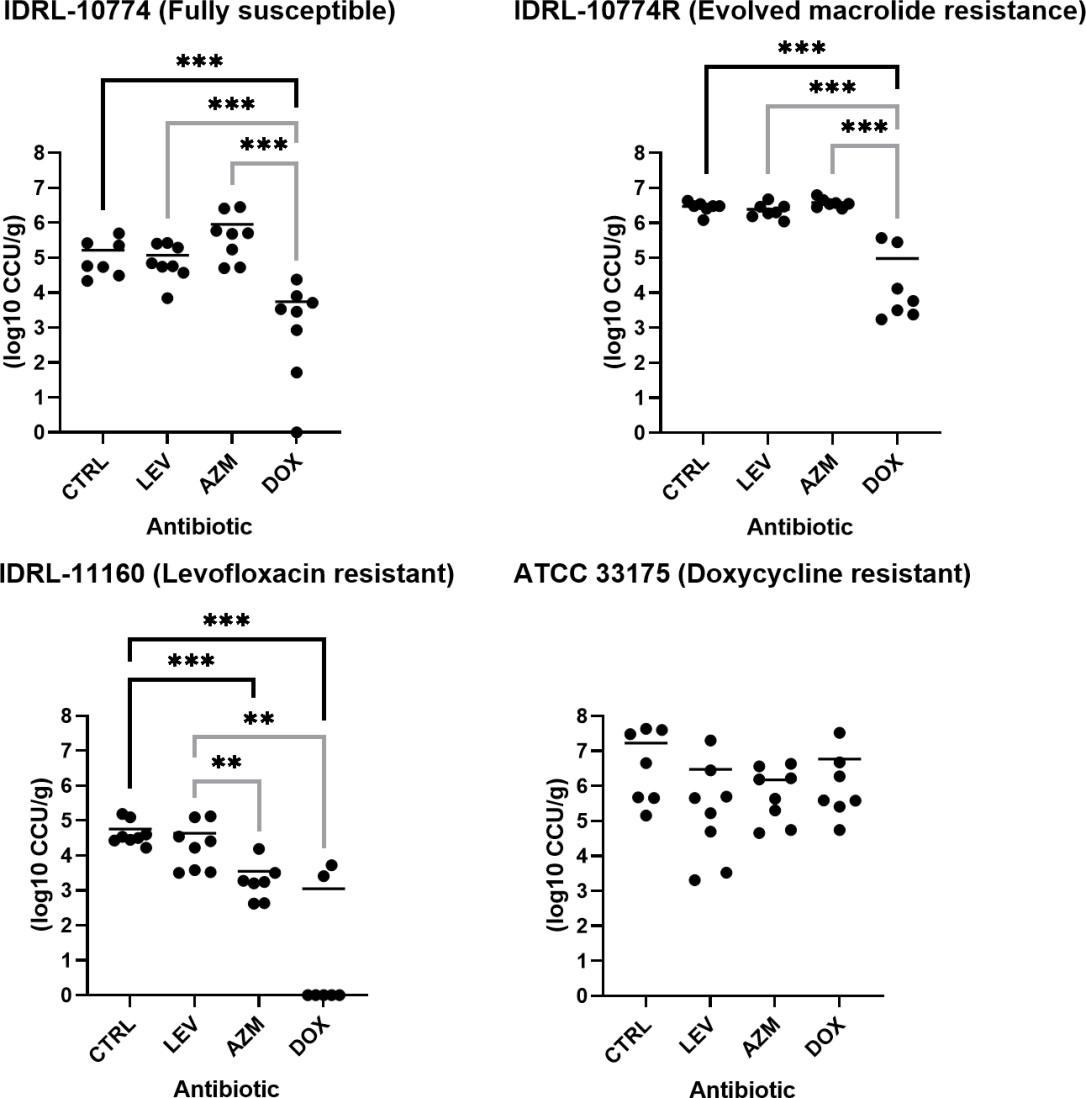
Antibiotic treatment of murine infection by antibiotic-resistant *Ureaplasma* isolates. Mice were treated with systemic azithromycin (AZM), levofloxacin (LEV), or doxycycline (DOX) 24 hours after infection with *Ureaplasma parvum* IDRL-10774 (susceptible to levofloxacin, azithromycin, and doxycycline), *U. parvum* IDRL-10774R (evolved macrolide resistance, susceptible to levofloxacin and doxycycline), *U. parvum* IDLR-11160 (levofloxacin-resistant, susceptible to doxycycline and azithromycin), or *Ureaplasma urealyticum* ATCC 33175 (doxycycline-resistant, susceptible to levofloxacin and azithromycin). Lung bacterial loads (log_10_ CCU/g) were quantified 18 hours after treatment. Each treatment group was compared to no-treatment controls (black significance lines) and to one another (gray significance lines). Horizontal lines indicate the mean. Significance was determined via Mann–Whitney tests. *N* = 7–8. *P ≥ 0.05, **P ≥ 0.01, ***P ≥ 0.001.

To test the ability of antibiotics to treat established lung infections, mice were infected with *Ureaplasma* isolates resistant to one or no antibiotic. Twenty-four hours after initial infection, mice were reinfected and treated with systemic azithromycin, levofloxacin, or doxycycline. Eighteen hours following treatment, animals were euthanized, and lung bacterial loads were quantified. Results are shown in Fig. 3. Doxycycline reduced lung bacterial loads of mice infected with UP-FS (mean reductions, 1.47 log_10_ CCU/g *versus* controls; *P* = 0.0006), UP-AzmR (mean reductions, 1.49 log_10_ CCU/g *versus* controls; *P* = 0.0003), and UP-LevR (mean reductions, 1.71 log_10_ CCU/g *versus* controls; *P* = 0.0003), but not UU-DoxR. Apart from doxycycline, azithromycin reduced the lung bacterial load of UP-LevR (mean reductions, 1.21 log_10_ CCU/g *versus* controls; *P* = 0.0003). No other significant reductions were observed in any group. Only doxycycline reduced infection below LOD for UP-FS (1/8 below LOD) and UP-LevR (5/7 below LOD). No hyperammonemia was detected in any animal.

## DISCUSSION

*Ureaplasma* infections are linked to hyperammonemia syndrome in lung transplant recipients and can be mitigated by utilizing *Ureaplasma*-targeted antibiotics (14–17). However, the ideal prevention and treatment regimens remain undefined. In this study, the prophylactic and treatment efficacy of doxycycline, azithromycin, and levofloxacin against both resistant and susceptible *Ureaplasma* isolates was assessed in an immunosuppressed murine infection model.

For both prophylaxis and treatment, doxycycline was the most effective antibiotic, significantly reducing lung infection load in all isolates except for the doxycycline-resistant isolate (UU-DoxR). Doxycycline was also the most effective prophylactic antibiotic. Encouragingly, in studies that have surveyed *Ureaplasma* resistance, doxycycline/tetracycline resistance has typically been low (20, 30–33), making empiric doxycycline a promising and broadly applicable antibiotic option. Levofloxacin provided effective prophylaxis for IDRL-10774, with and without evolved macrolide resistance (UP-FS/UP-AzmR), but not for the levofloxacin-resistant isolate (UP-LevR) or, interestingly, the doxycycline-resistant isolate (UU-DoxR), and was not active in treating established infection by any study isolate. Azithromycin was only active as a prophylactic agent against UU-DoxR and as a treatment of infection by UP-LevR. Notably, both azithromycin and levofloxacin, while not being as effective as doxycycline in terms of reduced bacterial burden, did prevent infection in multiple animals (levofloxacin preventing half of infections with UP-FS and UP-AzmR and azithromycin preventing a third of infections with UP-FS).

Various treatments have been used for *Ureaplasma*-associated hyperammonemia in lung transplant recipients (34). For human extragenital infections in general, tetracyclines such as doxycycline, macrolides such as azithromycin, and fluoroquinolones such as moxifloxacin (400 mg orally or IV once daily) (35) or levofloxacin (500 mg orally or IV once daily) (35) have been recommended as first-line treatment (35). While levofloxacin was more effective than azithromycin in this study, fluoroquinolone resistance seems to be more common than macrolide resistance (31, 33, 36–38); however, resistance rates to the studied antibiotic classes vary from study to study, likely due to differences in susceptibility testing methods and regional variation. Based on the results of this study, doxycycline (200 mg loading dose, then 100 mg PO or IV twice daily) (35) could be favored among first-line options.

Given the risks of inherent and acquired resistance, combination therapy may be reasonable (18, 34, 39, 40). In a recent meta-analysis of cases of mollicute-associated hyperammonemia syndrome in solid organ transplant recipients, Barnes et al. found that a combination of fluoroquinolone and doxycycline was the most commonly used antibiotic treatment (34). Based on data presented here and reported resistance patterns (20, 30–32), inclusion of doxycycline should be considered, unless, of course, resistance is known to be present.

As for prophylaxis, data are more scarce. Some centers administer empiric therapy at the time of transplant, particularly if the donor is known to be colonized with *Ureaplasma* species. For instance, recipients at one center routinely receive levofloxacin (500 mg daily) and azithromycin (500 mg daily) starting on postoperative day 0 until BAL testing returns negative. If BAL results are positive, treatment is continued for a minimum of 14 days (17). Another prophylactic strategy involves collecting donor BAL samples intraoperatively or within 24 hours of transplantation, followed by high-dose azithromycin administration to the recipient for 5 days, or until BAL results are available. If the BAL tests positive, recipients pre-emptively receive levofloxacin and doxycycline. Otherwise, they transition to low-dose azithromycin, with doxycycline reserved for patients with prolonged QTc intervals (34). Prophylaxis of donors has also been suggested (18).

This study has several limitations, the first of which is the use of an animal model that represents lung infection in immunosuppressed mice rather than a lung transplant model. Differences in post-lung transplant recipient physiology, such as decreased blood flow and ischemia in transplanted lungs (41), could change antibiotic pharmacokinetics at infection sites. Further, pharmacokinetics and pharmacodynamics differ between humans and mice due to variations in physiology, metabolism, and drug-processing enzymes, which can impact drug absorption, distribution, clearance, excretion, and overall effectiveness (28, 42). The human *Ureaplasma* species studied here, *U. parvum* and *U. urealyticum*, also do not survive well in this mouse model, necessitating re-inoculation of the lungs (15, 16, 24). As such, the treatment protocol included a reinfection step 24 hours after initial infection (at the time of antibiotic dosing), which is not a clinically relevant scenario. *Ureaplasma* survivability in the model also necessitated a shorter treatment duration, as reinfection was not logical in the prophylaxis arm of the study, and not practical in the treatment arm, given that new infections would be continuously retreated 24 hours after they were administered, instead of treating a single infection over the course of days to weeks, as is done clinically. There was also variability in infection success between the isolates, despite administration of the same inoculating dose, and as such, lung infection load in the control (no treatment) groups varied between isolates, which could affect antibiotic efficacy, and may explain the success or lack thereof of certain antibiotics against isolates that carry no resistance to them. Additionally, although blood ammonia was measured, no hyperammonemia was detected, likely due to the transient nature of the infection. Lastly, the immunosuppression protocol utilized mimics maintenance immunosuppression in lung transplant recipients and not induction immunosuppression, such as the use of antibodies that greatly and immediately deplete lymphocytic activity (43).

### Conclusion

In this study, doxycycline outperformed azithromycin and levofloxacin as a prophylactic and treatment agent for experimental *Ureaplasma* murine lung infection, except against a doxycycline-resistant isolate. While levofloxacin and, to a lesser extent, azithromycin showed some prophylactic benefit, doxycycline’s lower resistance rate across *Ureaplasma* isolates highlights its potential as a prophylactic and treatment agent for *Ureaplasma*-induced hyperammonemia syndrome in lung transplant recipients.

## Data Availability

Data from this study is available upon request from the corresponding author.
